# Challenges and visions for managing pain-related insomnia in primary care using the hybrid CBT approach: a small-scale qualitative interview study with GPs, nurses, and practice managers

**DOI:** 10.1186/s12875-021-01552-3

**Published:** 2021-10-20

**Authors:** V. E. J. Collard, C. Moore, V. Nichols, D. R. Ellard, S. Patel, H. Sandhu, H. Parsons, U. Sharma, M. Underwood, J. Madan, N. K. Y. Tang

**Affiliations:** 1 Department of Psychology, University of Warwick, Gibbet Hill Road, Coventry, CV4 7AL UK; 2 Clinical Trials Unit, Warwick Medical School, University of Warwick, Coventry, CV4 7AL UK; 3 University Hospitals Coventry and Warwickshire NHS Trust, Coventry, CV2 2DX UK; 4 University/User Teaching and Research Action Partnership, University of Warwick, Coventry, CV4 7AL UK

**Keywords:** Chronic pain, Insomnia, Sleep, Cognitive behaviour(al)therapy (CBT), Psychological treatment, Hybrid CBT, Patient and public involvement (PPI), Primary care, Thematic analysis, Pain management

## Abstract

**Background:**

Chronic pain and insomnia have a complex, bidirectional relationship – addressing sleep complaints alongside pain may be key to alleviating patient-reported distress and disability. Healthcare professionals have consistently reported wanting to offer psychologically informed chronic pain management at the primary care level. Research in secondary care has demonstrated good treatment efficacy of hybrid CBT for chronic pain and insomnia. However, primary care is typically the main point of treatment entry, hence may be better situated to offer treatments using a multidisciplinary approach. In this study, primary care service providers’ perception of feasibility for tackling pain-related insomnia in primary care was explored.

**Methods:**

The data corpus originates from a feasibility trial exploring hybrid CBT for chronic pain and insomnia delivered in primary care. This formed three in-depth group interviews with primary care staff (*n* = 9) from different primary care centres from the same NHS locale. All interviews were conducted on-site using a semi-structured approach. Verbal data was recorded, transcribed verbatim and analysed using the thematic analysis process.

**Results:**

Eight themes were identified – 1) Discrepant conceptualisations of the chronic pain-insomnia relationship and clinical application, 2) Mismatch between patients’ needs and available treatment offerings, 3) Awareness of psychological complexities, 4) Identified treatment gap for pain-related insomnia, 5) Lack of funding and existing infrastructure for new service development, 6) General shortage of psychological services for complex health conditions, 7) Multidisciplinary team provision with pain specialist input, and 8) Accessibility through primary care. These mapped onto four domains - Current understanding and practice, Perceived facilitators, Perceived barriers, Ideal scenarios for a new treatment service – which reflected the focus of our investigation. Taken together these provide key context for understanding challenges faced by health care professionals in considering and developing a new clinical service.

**Conclusions:**

Primary care service providers from one locale advocate better, multidisciplinary treatment provision for chronic pain and insomnia. Findings suggest that situating this in primary care could be a feasible option, but this requires systemic support and specialist input as well as definitive trials for success.

**Supplementary Information:**

The online version contains supplementary material available at 10.1186/s12875-021-01552-3.

## Introduction

In the UK, chronic pain constitutes a substantial burden to primary care in terms of appointment presentation and direct cost. Accounting for 4 million general practitioner (GP) appointments per annum with total healthcare costs approximately double that of individuals presenting without chronic pain [1, 2]. Typically, people with chronic pain present with multiple comorbidities. However, insomnia is consistently cited by patients as the most common and disruptive with up to 90% reporting symptoms at clinical levels [3–13]. Insomnia alone is a risk factor in the development of many adverse health outcomes, including hypertension, cardiovascular disease, diabetes, respiratory diseases, and increased mortality [14–16]. Poor sleep can be a driver of persistent pain alongside associated distress and disability [17–25].

People with chronic pain have long highlighted better sleep as a key treatment outcome [26–28], but its delivery remains peripheral in pain management programmes (PMPs) even though more than one meta-analyses have now shown beneficial treatment effects of non-pharmacological treatments of insomnia for people with chronic cancer and non-cancer pain [29, 30]. In primary care, where a considerable amount of day-to-day chronic pain and insomnia management occurs, pharmacological interventions persist as main treatment options, despite limited evidence supporting efficacy and safety beyond 6-12 months [31]. Reported undesirable effects after short-term use include excessive daytime somnolence, increased risks of falls, varied cognitive impairments and road traffic accidents [32–35]; indeed, side effects after long term usage include increased risk of respiratory depression, dementia, and mortality [36–40]. As polypharmacy increases, these effects become more pronounced, especially with combined use of opioids and benzodiazepines which is considered to have contributed to the sharp increase in deaths from accidental prescription drug overdose [41]. Moreover, clinical guidance and review evidence clearly warn about the potential of addiction to insomnia medications, due to the rapid development of tolerance and withdrawal symptoms during dose reduction [42–46] Psychological treatments that simultaneously target chronic pain and insomnia offer promising alternatives [47].

A 2007 evaluation of a talking therapy modified for people with pain-related insomnia presenting at a hospital clinic [10] provided a potential treatment model to adapt into primary care. The intervention simultaneously tackled chronic pain and sleep with select components of CBT-I alongside CBT-P interventions targeting the maintenance processes of chronic pain. This hybrid approach was delivered on an individual basis for 4 weeks via weekly two-hour sessions, the treatment dosage of which approximated the optimal dose recommended for CBT-I [48, 49] within stepped care models [50]. Post-treatment, when compared with a symptom-monitoring control procedure, the intervention was associated with greater improvement in sleep. Pain intensity did not change; but hybrid CBT was associated with greater reductions in pain interference, fatigue, and depression relative to the control [10].These findings correspond with those from a handful of other randomised control trials conducted in the USA and Spain with hybrid CBT showing better treatment outcomes, than when CBT-I or CBT-P are offered alone [51–55]. Hence, the addition of CBT-I is necessary for optimal management of pain and pain-related insomnia. Study authors have recently completed a linked feasibility trial to explore how such a service could be delivered in primary care in the UK [56]. This also comprised four weekly, individual 2 hour sessions to reflect optimal treatment dosage for CBT-I and maintain the level of treatment content (1 hour CBT-I, 1 hour CBT-P) whilst minimising travel burdens and treatment durations, of which may hinder engagement in this patient group [56, 57].

Here we report an interview study of primary care staff involved in the feasibility trial, the main aim of which was to inform planning for a future definitive study. However, the content of these discussions spoke to wider issues pertinent for managing pain-related insomnia in primary care. Via thematic analysis [58], we aimed to generate a qualitative understanding of present challenges and possible solutions to providing hybrid CBT and psychological treatment in primary care settings.

## Methods

### Recruitment

Participating practices were geographically spread across Coventry and Warwickshire identified by the Local Comprehensive Research Network to ensure different demographics, practice settings, and experience with research participation. Practices had between 5000 and 8000 registered patients, with between two and six GPs. Practices’ demographic composition was widely varied (respective samples 2.1, 3.7 and 25.8% in each were of ethnic minority backgrounds). These practices also represented a wide spread of socio-economic backgrounds with respective scores of 8, 4 and 1 on the 1-10 Index of Multiple Deprivation (IMD), with lower scores indicating higher levels of deprivation [59, 60].

The IMD was used for two reasons; first, to gain a robust sense of relative deprivation of the populations served by our participating practices. Second, as it utilises seven domains known to impact deprivation levels (Income; Employment; Education; Skills and Training; Health and Disability; Crime; Barriers to Housing Services; Living Environment), the resultant data gives a more rounded understanding of potential complexities and barriers (or lack thereof) to a range of questions regarding access to healthcare and relative needs. Previous research has linked indicators of deprivation, such as lower social economic status and other associated factors with poorer sleep and pain outcomes, emphasising the reach of these issues in each of our practice’s served populations [61, 62].

### Sample

The sample comprises nine primary care service providers (one GP, one nurse, and one practice manager from each participating practice). Three separate group interviews by practice were conducted. One practice manager opted to sit in as an observer of the discussion instead. Participants were purposively recruited via email through the linked feasibility trial. Inclusion of medical, caring and management perspectives was to gain a more thorough understanding of practices’ day-to-day operations.

### Interviews and procedure

All group interviews were conducted on-site in each practice, ranging from 30 to 60 min in duration. Participants were interviewed once post-trial, within 3 months of recruitment completion (May and June 2016, February 2017). A semi-structured interview schedule with 10 seed questions was used to anchor discussion, whilst allowing for organic interactions, i.e., interactions between the interviewer and participants, as well as among participants allow for spontaneity and natural tangents in conversation (Table 1). Digression from the schedule was also allowed, e.g., for the interviewer to follow up on a response with prompts or questions for clarification where appropriate or for the interviewees to discuss related topics. The seed questions were devised during the planning stage of the linked feasibility trial [56] through multiple consensus meetings between research team members to aid with overall process evaluation.

**Table 1:** Interview seed questions

1.	If a patient consults with chronic pain and sleep problems what would be your normal practice?
2.	Are there any existing services you may refer people with chronic pain and/or sleep problems to?
3.	Do you feel equipped to treat patients with chronic pain and sleep problems?
4.	Do you think there are other ways we could be helping these people?
5.	If we developed a service for patients with chronic pain and insomnia, what might it look like?
6.	Where would it sit as a service primary care/ secondary care or somewhere else?
7.	Why did your practice decide to help with this research?
8.	Do you see a need for the treatment we are offering?
9.	What do you see as the possible impact/implications of the study?
10.	Would you like to see the service that we are offering continue?

One interviewer conducted each of the group interviews (VN) and made field notes during and immediately after interviews. These did not form part of data analysis but helped keep track of key discussion points and areas for further exploration. Interviews were tape-recorded and transcribed verbatim for later analysis (VN, CM, VEJC). VN was a Research Fellow on the study’s team with extensive experience in mixed-method methods approaches to health service research and working in the National Health Service (NHS) as an allied healthcare professional. Data transcription was supported by the project co-ordinator (CM) and a psychology PhD researcher affiliated with the study (VEJC), who also observed one of the group interviews. These three researchers did not know the interviewees nor have established links with the practices prior to the study.

The study protocol was reviewed and granted favourable ethical opinion by the Solihull NHS Research Ethics Committee (REC NRES reference number: 14/WM/1053). Written informed consent was obtained from each participant prior to group interview commencement. Assurances of confidentiality and omissions of potentially identifiable data were made alongside the option for participants to decline any or all the data usage in subsequent analysis.

### Data analysis

Three group interview transcripts formed the data corpus. Thematic analysis was employed, guided by six key processes as set out by Braun and Clarke (2006) [58]. Findings are presented with exemplar quotes that substantiate interpretation. Exemplar quotes were chosen via an iterative process between VEJC, CM, and NT during the latter stages of analysis. The process was then repeated with the wider study team during report production to certify that the chosen quotes best corroborated the thematic narrative. Any quotes which team members felt did not fully reflect or support the narrative were discussed and rectified. All quotes are accompanied by an anonymised practice identifier, such that the extent to which each practice is represented in the results is transparent.

Two researchers (CM and VEJC) performed the analysis independently using NVivo, as a means to further establish rigour and credibility, and allow for unanticipated independent insights [63, 64]. Initial codes and themes were discussed between them to resolve any differences in opinion. The agreed codes and themes were then discussed and re-organised with senior team members, who served as content-experts to contextualise generated themes in relation to previous research and clinical practice experience (DE, VN & NT). Themes were derived from the data within parameters discussed in the interview schedule. Themes that ‘survived’ this stage of analysis were then sent to all group interview participants for comments, as a further step to enhance credibility and authenticity of the analysis [63]. As an additional measure for credibility, follow-up meetings (conducted November 2018) with NT and VEJC were offered to all three practices as an opportunity to discuss and review initial findings. Two face-to-face meetings took place, each was approximately one-hour long. One practice could not commit to a follow-up meeting due to key personnel change and heavy workloads, however, did provide email confirmation that they were satisfied with initial analysis and direction.

## Results

**Fig. 1:**
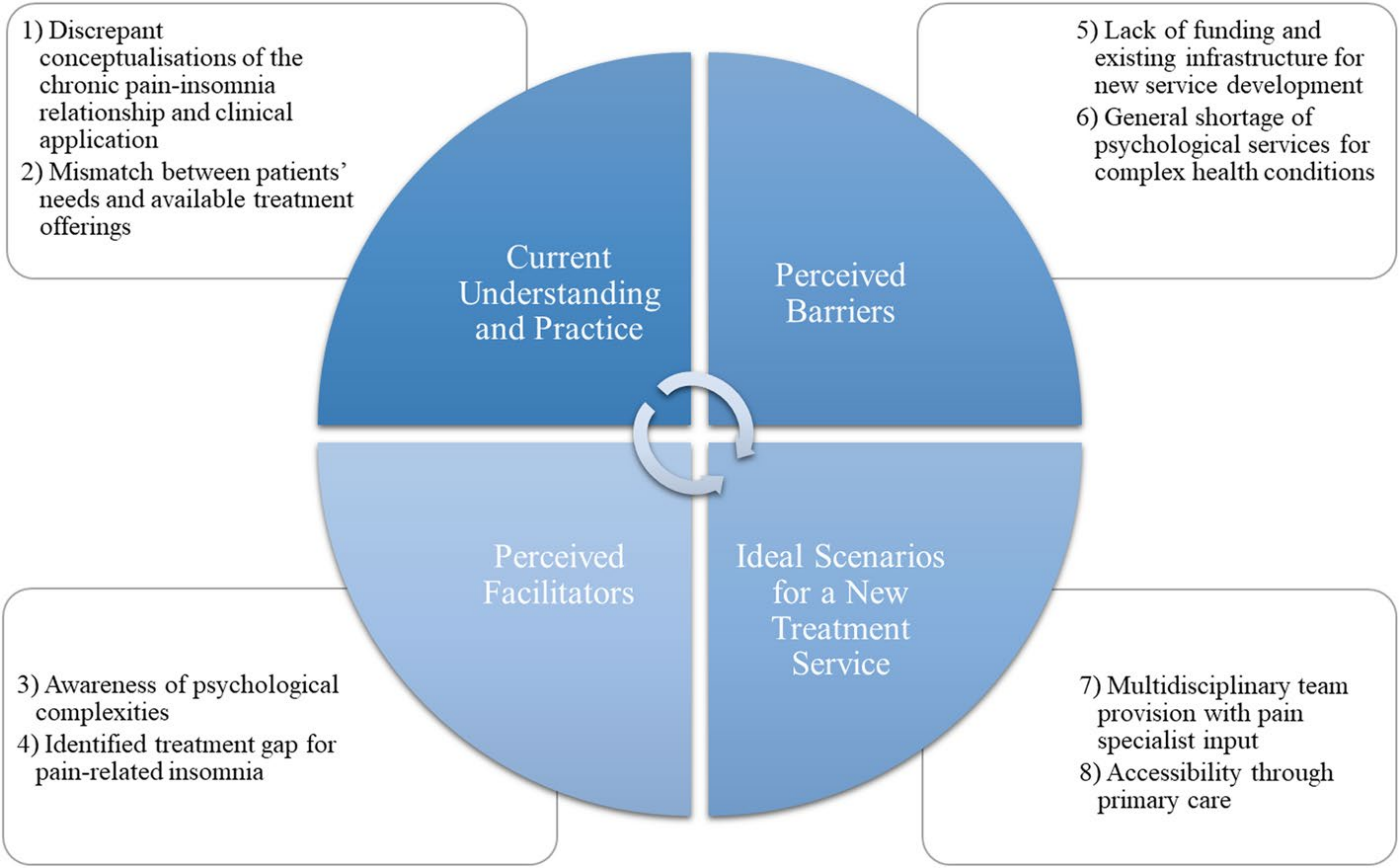
Summary of eight extracted themes mapped onto four domains embedded in interview structure

In total, eight themes were identified, which mapped onto four broad domains drawn out from the interview schedule. Themes and domains together contextually presented the issues of concern and were seen as functionally linked whereby the dynamics of perceived facilitators and barriers inform whether current practice can evolve into ideal treatment scenarios (Fig. 1).

### Current understanding and practice

#### Discrepant conceptualisations of the chronic pain-insomnia relationship and clinical application

An important thread running through all discussions was how participants understood the chronic pain-insomnia relationship and subsequent implications to treatment. There were nuanced differences between GPs in terms of understanding and treating patients presenting with pain and pain-related insomnia. Such that, whilst some providers considered pain-related insomnia as a symptom of chronic pain, others showed appreciation that its presentation could have a reciprocal impact on pain and would prescribe or prioritise treatment accordingly.

- ***A) Practice A:*** “Well to try and sort out what’s the cause of the chronic pain and try to alleviate that and see if that improves their sleep problems…Undoubtedly pain affects sleep long term […] pain will affect sleep over a long period of time…”.
- ***B) Practice B:*** “So if I had a patient who had insomnia symptoms as well I would strive to treat the insomnia as well as the pain because what you find is insomnia can exacerbate the pain and the pain can exacerbate the insomnia. So for us it’s dual approach in treatment.”

These variations in views or working hypotheses ultimately fed into differences in prescription and treatment plans specific to sleep. Some showed high confidence of certain drugs’ effectiveness in aiding sleep whilst others showed reluctance in using hypnotics the primary intervention for managing insomnia. It is often the case that hypnotics are the first and only intervention proposed however, as per clinical recommendations, should not be the primary intervention for people presenting with sleep disturbances [65]. These differences were embedded within our sample’s different treatment heuristics and practice policies.

- ***C) Practice B:*** “The sort of chronic neuropathic pain drugs especially Amitriptyline is the favoured one which we use a lot in terms of trying to ensure patients get quality sleep but also trying to manage the pain…”.
- ***D) Practice C:*** “As for us prescribing anything for the sleep which is discouraged in our practice. We don’t start anyone on any form of hypnotics or sleeping tablets in this practice”.

The surge in research and clinical interest regarding sleep and pain’s bi-directional relationship has only recently occurred [66, 67]. Thus, evidence demonstrating a stronger influence from sleep disturbances on pain than from pain to sleep and therapeutic effects of insomnia treatment for pain management and quality of life may not have fully translated to clinical practice yet. Interestingly, participation in research was cited as a practical way to mitigate this.

- ***E) Practice C:*** “…sometimes you want to review your practice and see what other people are doing and where different people or different health professionals are coming from. That gives their perspective too and also when there is…when you get to know the results of the study then… you try to change your practice.”

#### Mismatch between patients’ needs and available treatment offerings

Discussions show that medical staff’s treatment decisions were strongly motivated by the idea of targeting an “underlying” cause.

- ***F) Practice B:*** “When the patient presents with insomnia as their overriding issue then clearly we try to identify the underlying cause of what that might be. It could be due to a chronic back problem it could be down to a rheumatological issue…”

Initially using a unimodal treatment approach to deal with comorbid chronic conditions like pain and pain-related insomnia that share a bi-directional relationship and a biopsychosocial underpinning [68, 69], give way to at least two inherent dilemmas. First, initial applications of ‘underlying cause first’ treatment logic would restrict the treatment focus to the pain. This might impede treatment progress as chronic pain is, by definition, intractable and patient’s quality of life is typically determined by more than just pain severity. Second, for cases that were thought to have a strong ‘social’ or ‘psychological’ element, medical staff felt they would ultimately struggle to offer what the patient actually needed considering that the treatment options at their disposal were mainly pharmacological in nature.

- ***G) Practice C:*** “I often feel patients are caught in a system now whereby sometimes all the issues aren’t addressed because a big part of pain is psychological. And I feel the psychological element of pain is never investigated...We don’t have like a pain service, purely psychological services. We have the chronic pain service which meets the pain issue with …medication or with injections…We have got the [Improving Access to Psychological Therapies] IAPT service…But I don’t think they’re so geared up to treating pain-related psychological issues they may be more geared up towards the depression…”
- ***H) Practice C:*** “… People with chronic pain for example are living in flats where there are no lifts or their housing problems or transportation …These are certain small things which may not affect general population but may affect these small cohort of patients more…”

### Perceived facilitators

#### Awareness of psychological complexities

Overall, there is some shared understanding that pain is a multidimensional, individuated experience and that this should be reflected in treatment provision. Hence, participants welcomed the idea of a new service to simultaneously manage chronic pain and insomnia. Particularly, that the psychological elements accompanying chronic, complex health conditions should form part of treatment guidance. From our sample, it seems that practices are keen to shift towards a more integrated, biopsychosocial formulation supporting self-management in chronic pain and pain-related insomnia in primary care.

- ***I) Practice A:*** “…within primary care…there is a very big move away from using medicines to treat insomnia…I don’t necessarily think drugs are always the answer because drugs tend to cover symptoms up, particularly with pain. And drugs have lots and lots of side effects too, so I like the idea of something which is non-drug related ideally it should also empower the patient to take their share of responsibility for what is a long-term condition…”

#### Identified treatment gap for pain-related insomnia

Enthusiasm for a new psychologically informed service for pain-related insomnia provided under one roof, in part, stemmed from a clear treatment gap, where staff saw an ever-increasing demand in their practice populations and a general absence of long-term meaningful psychological support for this patient group.

- ***J) Practice A:*** “I think it’s a big population group [people with both pain and insomnia]. And I think we’ve probably got tens of patients who’d benefit from it. There are 36 practices…so you’re probably looking at hundreds of patients every year who’d need something like this. So it’s a big thing.”

However, discussions showed disparity between this awareness and the reality of what primary care staff could feasibly offer to patients. This fed into current management of these issues was considered a major burden to GP time, hence, costly.

- ***K) Practice C:*** “There’s not an awful lot of services for them really…If I saw somebody having chronic pain that was sleep-related, I wouldn’t know what to do.”
- ***L) Practice B:*** “…if we had a pain management service in house, I could probably reduce my consultations by probably 20%.”

Participants expressed that if the new treatment could help patients improve their quality of life, return to work, and reduce long-term utilisation of health services, demonstrating the resultant financial savings could be a key motivator of service development.

- ***M) Practice A:*** “Look at returning people to work…The longer people are off work the less chance they’ve got of going back…could be very cost-effective to invest in these people.”

### Perceived barriers

#### Lack of funding and existing infrastructure for new service development

Funding, location, and delivery seemed to be the most prominent issues associated with new service development. Although these barriers are not specific to the proposed new pain-related insomnia service development; their inclusion is necessary to corroborate similar barriers to treatment provision in other long-term, complex health conditions. Indicative of the economic climate at time of data collection, a major concern was if there was enough funding available to design and implement a new service. Participants highlighted that commissioning decisions were based on not just patient benefits but also financial savings, as money was ring-fenced for priority areas to fund services considered by NHS Clinical Commissioning Groups (CCGs) as ‘absolutely required’. Thus, the amount of money left to commission new services was considered relatively limited.

- ***N) Practice A:*** “…you’ve got a devil’s own job being able to demonstrate that, because at the moment in the NHS being able to demonstrate benefit doesn’t seem to be enough, you have to demonstrate financial savings.”
- ***O) Practice B:*** “…the difficulty is as far as the CCG’s position is concerned is [they’re] in a financially compromised position. They’re addressing services that they feel are absolutely required.”

Although patient demand is there, there is no established care pathway for accessing psychological treatments for chronic pain, even less so for pain-related insomnia. Considerations were given to how the proposed Hybrid CBT could be offered in primary or secondary care, to maximise treatment coherency and patient uptake. Each practice focussed on specific issues surrounding lack of general infrastructure, which may echo what they see as most troublesome for their patients.

#### General shortage of psychological services for complex health conditions.

Participants were aware of the fact that medical staff would not have the time and training to deliver the proposed new service, thus expressed concerns over a shortage of psychologists sufficiently trained to deal with patients who present with complex health conditions in existing primary and secondary care services.

- ***P) Practice A:*** “We haven’t really got access to anybody who’s specifically good with insomnia. ... we use IAPT for low level psychology problems that may be the kind of place you could refer somebody for insomnia, maybe. I don’t think anybody’s pulled the two things [chronic pain and insomnia] together….”

Certainly, there is awareness of current psychologically informed services dealing with insomnia, but not at the level of complexity required for pain-related insomnia. Moreover, potentially symptomatic of how pain and its comorbidities are dealt with, it does seem that even where there is psychological provision this is either a last resort or peripheral in current treatment plans.

- ***Q) Practice B:*** “… [the pain clinic] say there’s a psychotherapist or psychologist already working there, which I don’t think is being utilised really to its full effect or they’re being inundated…”

### Ideal scenarios for a new treatment service

#### Multidisciplinary team provision with pain specialist input

Regarding future treatment, all participants showed conviction in how they thought this should manifest to address patients’ needs. Whilst the treatment adopted in the current feasibility trial was offered as a stand-alone psychological intervention, there were strong feelings that future developments of such treatment should look at provision by a multidisciplinary team.

- ***R) Practice A:*** “…it would be a multidisciplinary service, it would probably have physiotherapists…osteopaths and chiropractors involved…I think also in there, I don’t know whether it needs a psychologist or whether it needs just counsellors but it needs somebody in there to look at the psychological aspects of the pain, its effect on insomnia and strategies that that person can use when its three o’clock in the morning…and they haven’t got any sleeping tablets or pain killers.”

Such that the nature of the service would be co-ordinated, with psychological and practical support for patients and an emphasis on promoting self-management.

- ***S) Practice C:*** “It has to be a multi-disciplinary approach with different health professionals specialising in different avenues for managing these patients, sit together and formulate a management plan.”

There was, however, less certainty with regards to where the new service should sit within existing treatment pathways. Discussed options included incorporating the service into current PMPs, IAPT services, and setting up a separate service specifically for pain and sleep management to be supported and shared by a collaborative GP network.

- ***T) Practice B:*** “You could align it with IAPT if you wish. But you’d need someone who has a specialist interest and understands pain in the essence…”

#### Accessibility through primary care

Regardless of where the service might sit within existing care pathways, one key requirement for such new treatment service was accessibility. This, combined with previous suggestions that the service should bolt on to the pain clinic’s multi-disciplinary team, brought forth debate as to whether the service would situate better within primary or secondary care from both conceptual and practice perspectives.

- ***U) Practice C:*** “…The first port of call for any patient is primary care…with the current economic climate if at all we set up a service which is going to affect the secondary care or hospitals more then I don’t think it will be a good use of NHS resources, so it has to be primary care.”
- ***V) Practice B:*** “Best centred here [primary care]. Because the demand is here….and based in practice…being local... A lot of them rely on buses and things like that. A lot, sorry quite a fair few don’t drive, especially the elderly patients…and financially, they’ve got to pay out to travel in as well.”

Even though differences exist between discussions, overall, a change in situation and access points from largely secondary to primary care level was considered necessary for optimal delivery and accessibility.

- ***W) Practice A:*** “It’s the kind of problem that should be sourceable in primary care but administratively I’m not sure how you would do that in each individual practice. So you probably need some kind of hub…”

Indeed, trade-offs in terms of practicality and current financial constraints were felt to impede creating new, or even altering current treatment pathways for pain-related insomnia patients.

## Discussion

We have identified primary care viewpoints from three practises in a single NHS locale on care provision for patients with pain-related insomnia, from their experiences as trial sites for a recent feasibility study [56]. Guided by a semi-structured interview schedule, our participants considered the current landscape of tackling pain-related insomnia in primary care and how it might be improved within the current parameters as well as ideal scenarios. Their viewpoints were contextualised through eight themes, situated within four broad domains that reflect the focus of investigation (Fig. 1). The overall sum of discussions speaks to a need for an integrated service supported by an established yet adaptive infrastructure in the care system. This coincides with the hybrid treatment concept [56, 57, 70] that surrounds the data corpus and recommended strategies on how this could be successfully implemented. Our subsequent discussion points are framed as starting points for future trials, taking into account that much of the components and questions for successful management are in flux.

### Is the proposed integrated service worth the challenges, and are these unique?

Implementing an integrated service in primary care will incur some challenges, namely that the appropriate infrastructure, is not yet in place to deliver such treatment for this patient group [39, 71]. Infrastructure is manifold in describing the sum of physical, technical and organisational components needed in the context of healthcare delivery [72]. This involves complex, highly integrated areas such as staff structure and training, framework situation and physical location, and treatment pathways/access points [73] when considering developing existing or new services. This is inclusive of a lack of a fully empirically validated protocol specific to pain patients presenting with pain-related insomnia [70] as well as a shortage of trained therapists to ensure appropriate delivery and follow-up [48, 50]. A previous qualitative systematic review synthesised these issues in relation to differing beliefs and expectations of both patients and GPs [74]. Specifically, in the context of beliefs about pain, treatment expectations, trust, and patient education. Findings also advocate a shift away from specialist services to treat pain in intensive, short time periods to community services that would provide ongoing holistic patient assistance including managing comorbidities [75]. However, there are numerous common barriers that contribute to this evidence-practice gap, inclusive of primary care staff’s misconceptions of addressing and implementing recommended treatment guidelines for best practice [76].

Arguably, these issues around infrastructure and implementation of best practice are not new nor specific to the management of pain-related insomnia. Given that comorbidity in chronic conditions and simultaneous presentation tends to worsen prognoses, intervention and management at the primary care level may lessen some of these associated challenges [77]. This may address previously stated undesirable outcomes associated with current pharmacological provision as ‘go-to’ treatment options for both chronic pain and insomnia management [29–38]. Moreover, there is some evidence demonstrating cost-effectiveness from successful collaborative care models in depression to promote biopsychosocial care models in primary care for chronic pain and insomnia [78]. Further integration and education of psychological care and PMPs in practices could also meet patients’ described needs and boost clinical adherence [74, 76].

### Is CPD a solution to address discrepancies in clinical practice?

Linked to infrastructure issues is information discrepancy surrounding how the pain-sleep relationship is conceptualised. This was reflected in differences between practices regarding how pain-related insomnia was described and treated whilst utilising secondary management models [79]. This may stem from clinical education around pain during medical training and post-qualification. Pain education is fragmented, falling short of what might be expected given the prevalence and burden of chronic pain across Europe [5]. Of 28 UK medical schools surveyed, only 11 had a dedicated pain medicine module, of which 4 offered it as a compulsory element [80]. None of which indicated addressing pain-related insomnia. However, evaluative research with the same dataset identified potential facilitators including reframing clinical management using a biopsychosocial framework [81]. Such was reflected in our participants’ considerations for improvements and ideal treatment scenarios. Since research has demonstrated the existence of a complex, bi-directional relationship which informs pain-related insomnia [17, 18, 20, 21, 25, 66, 82–84] further education and information exposure should focus on its clinical application and treatment in line with recommendations of integrated, multidisciplinary service provisions [85].

### Should a new service situate in primary or secondary care?

Even with differences between our participants about where such a service should situate in a treatment framework, there was clear support regarding psychological input and allied health professionals to run and manage it. Previous evaluation of nurse administered CBT-I for persistent pain has shown promising results through using relevant professionals to conduct effective delivery and specialist training of other relevant staff [86] as have other self-management programs for other chronic conditions such as arthritis, diabetes and asthma [87]. This would certainly fit with pain management recommendations advocating multidisciplinary, biopsychosocial approaches to chronic pain and insomnia management [88, 89]. However, according to the most recent National Pain Audit [90] there is an overall lack of multidisciplinary provision, even though 64% of NHS England services describe themselves as such, only 40% meet criteria (inclusion of a psychologist, physiotherapist and physician). Moreover, the Midlands (our practices’ geographical situation) are relatively poorly served with the least access to such provisions. Guidance on future planning of pain management service strongly encourage psychologically informed, multidisciplinary treatment of chronic pain to be available at the primary care level [91]. Thus, mirroring views expressed by our participants and policy guidance could be the basis for productive discussion on how to further research such provision in primary care.

### New service development or augment existing pathways?

By boosting psychological support in primary care through multidisciplinary avenues, there is potential to make sure that service provision is not solely GP dependent [1, 92, 93] and meet this patient group’s treatment expectations [94]. Interestingly, the clinical and cost effectiveness of sleep management in pan management programmes has been noted as a key issue to be addressed in future research as per the 2020 NICE draft guidelines [95]. Another option may be to augment treatment pathways and redistribute resources in PMPs or IAPT to meet described needs and provide a more cohesive, integrated service [96]. However, participants highlighted concerns that in their current forms these services are not adequately resourced to deal with the complex needs of pain-related insomnia. Indeed, there is review evidence that implementing clinical networks can improve overall healthcare delivery for complex conditions, thus bring together some currently disparate treatment avenues and build rationale for more specialist interventions to slot into some currently available services [96]. However, these issues warrant more definitive discussion between researchers, practitioners, and CCGs in the design of future trials or service development [91].

Recently, there has been some considerable progress made in terms of prescribing and delivering psychological interventions for insomnia using digital platforms. CBT-I is a manualised psychological intervention with a rich evidence base, as such is well placed as a scalable solution to bridge gaps in feasible delivery and access for people presenting with sleep complaints [97]. After rigorous testing and evaluation “Somryst” (previously known as Shut-i) has been given Food and Drug Administration (FDA) approval in the USA as a prescribed treatment for insomnia [98]. Similarly, in a UK/NHS context “Sleepio” has accrued an extensive evidence base supporting its acceptability, feasibility, and efficacy across a range of populations presenting with sleep complaints [97, 99–102]. Interestingly, there is a trial protocol to explore the efficacy of “Sleepio” for people with lower back pain which could prove fruitful and leverage the case made for addressing sleep complaints as a core component of PMPs [103]. Given the success of using digital platforms to disseminate CBT-I, it is possible that providing CBT-P and hybrid CBT through digital platforms could follow similar trajectories. The potential utility and cost-effectiveness of prescribing and delivering hybrid CBT using online platforms could help mitigate some of our participants’ mentioned barriers to treatment access. Central to these are related to an observed lack of trained therapists trained to understand and deal with the often-complex presentation of pain-related insomnia, as well as barriers to travel for older people, those on fixed incomes, frail, or with caring responsibilities.

### Limitations

As the purpose of these interviews was to inform the perceived feasibility of setting up a Hybrid CBT for pain and insomnia RCT [56], gaining insight from managerial, allied, and medical perspectives was deemed suitable. It is however acknowledged that there were possible demand characteristics on our clinical partners that might bias their views. Whilst there is no way to formally assess this, taking account of this possibility is important in the overall interpretation of these findings. Our sample size was small but in line with what was practically possible for a small-scale feasibility study [56]. The principle of ‘information power over data saturation’ has been applied here [104], whereby aim, specificity, theory, dialogue, and analysis are used to reflexively decide appropriate sample size [104, 105]. Information power suggests that the more information a given sample holds relevant to the study, then a lower number of participants is needed to develop new knowledge. Data saturation, in contrast is a process of constant comparison where every new observation is compared with previous analysis until no new information can be gained [106].

Differences in current practice between our practices may reflect differences in the operational characteristics as they were purposively sampled for the linked feasibility trial to consider geographical location, register size and population served, and experience in research participation. However, it is unclear exactly how these operational characteristics are shaping these practices’ differences in terms of chronic pain and insomnia treatments and would be an interesting focus for further research. Appropriate measures were taken to ensure rigour and transparency. Moreover, the interview schedule did not allow the examination of how GPs treat individual case presentations of chronic pain and/or pain-related insomnia. Thus, we could not delve into the nuances of current practice at the individual level. As a final point, because discussion was based on general recollection, there is always the possibility that these may not be wholly accurate representations of current practice.

## Conclusions

Tackling pain-related insomnia in primary care is a hugely complex issue where many components are in flux. This study provides some frontline experience about the current and future perceived landscape of treating pain-related insomnia in the context of primary care. These findings are consistent with other research and policy outlining the need to consider providing integrated, multidisciplinary care at the primary care level, with specialist input from relevant professionals, every step of the way, especially in the early stages of development [90, 95]. Moreover, the development of future trials and subsequent rolling out of tested interventions require systematic support with an existence of appropriate infrastructure. Although not definitive, these findings do offer possible directions for future research, clinical practice, and people-centred service development.

## Supplementary Information

**Additional file 1.**

## Data Availability

The dataset generated and analysed during the current study are not publicly available due to potentially identifiable information given by participants but is available from one of the senior authors (Prof Nicole Tang; n.tang@warwick.ac.uk) on reasonable request.

## Abbreviations

PMPs: Pain management programmes
CBT: Cognitive behavioural therapy
CBT-I: Cognitive behavioural therapy for insomnia
CBT-P: Cognitive behavioural therapy for pain
GP: General practitioner
NHS: National Health Service
CCG: NHS clinical commissioning groups
IAPT: Improving Access to Psychological Therapies
FDA: Food and Drug Administration
